# How should we evaluate the risk of bias of physical therapy trials?: a psychometric and meta-epidemiological approach towards developing guidelines for the design, conduct, and reporting of RCTs in Physical Therapy (PT) area: a study protocol

**DOI:** 10.1186/2046-4053-2-88

**Published:** 2013-09-26

**Authors:** Susan Armijo-Olivo, Jorge Fuentes, Todd Rogers, Lisa Hartling, Humam Saltaji, Greta G Cummings

**Affiliations:** 1 5-115A Edmonton Clinic Health Academy (ECHA), Outcomes Research Program University of Alberta, 11405 - 87 Avenue, Edmonton, AB T6G 1C9, Canada; 2 Department of Physical Therapy, Faculty of Rehabilitation Medicine, University of Alberta, 3-48 Corbett Hall, Edmonton T6G 2G4, Canada; 3 Department of Physical Therapy, Catholic University of Maule, Talca, Chile; 4 Center for Research in Applied Measurement and Evaluation (CRAME), 5-129 Education N University of Alberta, Edmonton, AB T6G 2G5, Canada; 5 Department of Pediatrics, Alberta Research Center for Health Evidence Faculty of Medicine, Dentistry University of Alberta, Edmonton, AB, Canada; 6 School of Dentistry, University of Alberta, Edmonton, AB, Canada; 7 Faculty of Nursing, University of Alberta Edmonton Clinic Health Academy, 11405-87 Ave, Edmonton, AB T6G 1C9, Canada

**Keywords:** Methodological quality, Risk of bias, Physical therapy, Meta-epidemiological, Factor analysis, Delphi procedure

## Abstract

**Background:**

Numerous tools and items have been developed in all health areas to assess the risk of bias of randomized controlled trials (RCTs). The Cochrane Collaboration (CC) released a new tool to assess bias in RCTs, based on empirical evidence quantifying the association between some design features and estimates of treatment effects (TEs). However, this evidence is limited to medicine and investigating a selected set of components. No such studies have been conducted in other health areas such as Physical Therapy (PT) and allied health professions. Evidence specific to the PT area is needed to understand and quantify the association between design features and TE estimates to inform practice and decision-making in this field. The overall goal of this project is to provide direction for the design, conduct, reporting and bias assessment of PT RCTs. We will achieve this through the following specific objectives and methods.

**Methods/Design:**

1) to measure the association between methodological components and other factors (for example, PT area, type of intervention, type of outcomes) and TE estimates in RCTs in PT, 40 randomly selected meta-analyses of RCTs involving PT interventions will be identified from the *Cochrane Database of Systematic Reviews*. Trials will be evaluated independently by two reviewers using the most commonly used tools in the PT field. A two-level analysis will be conducted using a meta-meta-analytic approach; 2) to identify relevant items to evaluate risk of bias of PT trials, an exploratory factor analysis (EFA) will be used to identify the latent structure of the items; 3) to develop guidelines for the design, conduct, reporting, and risk of bias assessment of PT RCTs, items obtained from the factor analysis and the meta-epidemiological approach will be further evaluated by experts in PT through a web-based survey following a Delphi procedure.

**Discussion:**

The results of this project will have a direct impact on research and practice in PT and are valuable to a number of stakeholders: researchers when designing, conducting, and reporting trials; systematic reviewers and meta-analysts when synthesizing trial results; physiotherapists when making day-to-day treatment decision; and, other healthcare decision-makers, such as those developing policy or practice guidelines.

## Background

Randomized controlled trials (RCTs), as well as systematic reviews (SRs) and meta-analyses (MAs) of these trials, are considered the gold standard to evaluate the efficacy and effectiveness of health care interventions [1]. The results from these studies are crucial for informing patients, clinicians and decision/policy-makers about the best treatments to be implemented to improve patient outcomes and the efficiency of the health care system. Assessing the methodological quality of trials is an essential component of systematic reviews, since only the highest quality evidence should be used to inform clinical and policy decisions. An accurate quality assessment is imperative in the synthesis of study findings in order to appropriately interpret results and effectively guide health care [2]. Quality assessment of studies has been used to: determine a minimum quality threshold for the selection of primary studies for systematic reviews, assist in determining the strength of inferences, and more importantly, to guide recommendations for future research, policy decisions, and clinical practice [2].

It has been demonstrated that different design features of a trial can have a substantial impact on estimates of treatment effects [3-7]. For example, it has been shown that inadequate allocation concealment or lack of double-blinding can overestimate treatment effects on average by 18% and 9%, respectively [4,6,7]. Other factors such as method of randomization [8,9], follow-up proportions, [10,11] and industry sponsorship [12,13] have also been shown to influence the results of trials. All of these factors lead to over-estimates of treatment effects, or bias, at the trial level, and can lead to biased or inaccurate results and conclusions in systematic reviews and meta-analyses [3,4,9,10,14]. This ultimately can have repercussions on decision-making and the quality of patient care since biased interventions (for example, interventions that are not as effective, ineffective, or perhaps even harmful) may lead to inappropriate expectations of impact, wasted resources and/or inappropriate uptake of evidence, respectively.

While the impact of primary trial bias on evidence synthesis has been recognized for years, the approach to quality assessment has been inconsistent and controversial [15]. Many tools (for example, Jadad, Chalmers, CONSORT, Delphi List, to name but a few) have been used to determine quality of RCTs in different health areas [15,16]. There is no agreement regarding which tools are optimal to accurately determine trial quality. Most tools have not been developed using scientifically rigorous methods, lack reliability and/or have not been fully validated [15]. In addition, the use of different tools for evaluating the quality of primary research can lead to different end results [17-19]. Thus, a clinical trial may be rated on a quality scale disparate by different measurement tools. This discrepancy in the evaluation of the quality of research may skew interpretation, reporting, and as a result, could potentially impact recommendations for clinical care. Finally, the tools include different items, some of which relate more to the detail of reporting rather than methodological quality.

As a result of these shortcomings with existing tools and methods for quality assessment, there has been a shift in the traditional scoring approach to the assessment of trial quality. Instead of examining trial quality with tools that have not been validated and often use composite scores, the assessment of 'risk of bias’ was proposed in 2008 by the Cochrane Collaboration, one of the most important and influential groups working on evidence based practice worldwide [20]. The Risk of Bias (RoB) tool was developed based on a growing body of empirical evidence quantifying the association between certain design features and estimates of treatment effects (TEs). The RoB tool includes six domains: sequence generation; allocation concealment; blinding; missing outcome data; selective outcome reporting; and 'other sources of bias’ (for example, early stopping for benefit, design-specific features such as adequate wash-out period in cross-over trials). Other methodological components within the RoB tool as well as other components that have traditionally been used to determine trial quality in health research have not been investigated; hence the evidence base is limited and incomplete. Furthermore, recent research [5,19] recommends further testing of the RoB tool to gain a better understanding of its psychometric properties, as well as to validate the tool in a wider range of research fields. Additional information will help users in applying and interpreting the results of the RoB tool.

Most of the empirical evidence regarding the relationship between trial components and treatment effect estimates comes from RCTs in the area of medicine and is based only on dichotomous outcomes [4,6,7]. No such studies have been conducted in other health areas such as the allied health professions, including physical therapy (PT). RCTs conducted in PT have unique features compared to drug trials conducted in medicine. PT interventions are classified as complex interventions [21] comprised of diverse facets that may affect the trial results, such as the type of therapy and its intensity, a standardized or individually tailored approach, and the skills and experience of the therapists. In addition, because of the nature of PT interventions (for example, manual therapy, exercises), blinding of the therapists and or the patients is not always possible. Appropriate blinding of participants and all key study personnel, therefore, is unlikely to be accomplished for most PT trials; however, blinding of outcome assessment has been used as a proxy quality measure without validation. Therefore, it is necessary that empirical evidence is performed and expanded in the area of PT and allied health professions in order to determine the factors that affect treatment effects estimates in these trials to provide accurate results to the clinical community. In addition, this information is urgently needed to develop guidelines for designing, conducting, and implementing PT trials as well as providing clear benchmarks to assess the risk of bias of PT trials in systematic reviews and meta-analyses and ultimately the strength of evidence for decision-making.

Therefore, the overall goals of this project are to provide direction for the design, conduct, and reporting of allied health RCTs, especially Physical Therapy (PT) and provide clear benchmarks for evaluating the RoB of RCTs in this area.

We will achieve this through the following specific objectives:

1) To measure the association between methodological components and treatment effects (TE) estimates in RCTs in PT;

2) To determine the influence of other factors (for example, clinical area, type of treatment, type of outcomes) on TE estimates;

3) To identify the underlying component structure of items to evaluate risk of bias of PT trials;

4) To identify relevant items to evaluate risk of bias of PT trials through experts’ opinion;

5) To develop guidelines for the design, conduct, reporting, and implementation of PT RCTs and provide clear benchmarks for evaluating the RoB of PT RCTs.

## Methods/Design

This Research protocol has been approved by the Ethics Board of the University of Alberta (Pro00038172).

### Objectives 1, 2 and 3

#### Study search

Meta-analyses and their RCTs will be obtained by searching the *Cochrane Database of Systematic Reviews* (meta-analyses) using the words physical therapy or physiotherapy, rehabilitation, exercise, electrophysical agents, acupuncture, massage, Transcutaneous Electrical Nerve Stimulation (TENS), interferential current, ultrasound, stretching, chest therapy, pulmonary rehabilitation, manipulative therapy, mobilization. Meta-analyses and their trials will be included if: 1) they included at least five RCTs comparing at least two interventions, at least one of which is currently or potentially part of PT practice according to the World Confederation for PT (WCPT) [22]; 2) the interventions in the trial will be applied to human subjects who were representative of subjects to whom the intervention might be applied in clinical PT practice; 3) the allocation of subjects to interventions was random or reported to be random; and 4) the authors provided quantitative data of treatment estimates.

#### Study selection

Two reviewers (physical therapists, both with at least 18 years of experience in physical therapy field and research) will independently screen the abstracts of the meta-analyses found in the Cochrane database and will analyze all papers initially selected by the abstract or title using the pre-defined inclusion/exclusion criteria (described above). Each criterion will be graded on a yes/no basis. Discrepancies between reviewers regarding inclusion will be resolved through consensus or in consultation with a third reviewer.

#### Sample size

Most meta-epidemiological work carried out until now has not formally performed sample size calculations since they have been exploratory in nature. However, according to a recent report, [23] most of these studies are under powered due to the small magnitude of the differences between trials with and without the quality domain, small sample sizes, and the high heterogeneity of the datasets. Based on this, the authors recommended assembling a homogenous set of meta-analyses and trials in a specific area of research and to have a larger number of trials included to increase the power of the study. Since this study is prospective, the magnitude of the difference between trials conducted with certain domains of trial quality and those that do not, is not known. However, it could be anticipated that a difference in effect sizes of at least 0.15 between trials with and without quality domains, as reported in previous meta-epidemiological work, would be obtained [11,24]. This magnitude of difference has been argued to correspond to one quarter to one half of a typical treatment effect found for interventions in areas similar to physical therapy. Thus, this difference would be relevant to the field. Therefore, sample size calculation for this project will be based on the following: information in a recent report, [23] research team, human resources, project budget, similar studies involving a continuous outcome, [11,24,25] and based on a search regarding possible meta-analyses from the Cochrane Collaboration to be included with outcomes of interest. We will target a number of 400 trials included in 40 meta-analyses approximately. This number of trials is almost double the number of trials included in previous meta-epidemiological work using continuous outcomes and/or evaluating interventions, and outcomes that could be comparable to the area of the PT.

A unique code generated by the Reference Manager bibliographic program will be assigned to each meta-analysis and trial that meets the inclusion criteria. This code will be used to randomly select the studies to be analyzed for this project and also to randomize the order of evaluation. The first author will randomly select each of the meta-analyses (MA) to be included, and accompanying trials, by drawing the code of the selected MA from an opaque envelope.

#### Data extraction and quality assessment

All of the trials will be evaluated independently by two reviewers using seven tools with their items (that is, Delphi List, PEDro, Maastricht, Maastricht-Amsterdam List, Bizzini, van Tulder and Jadad) that are most commonly used or most validated in the PT field [15]. Altogether 45 different items obtained from the seven tools will be used to evaluate the trials included for analysis in this study. All reviewers will receive the same training and guidance and will be provided with standardized guidelines to assess and to use all 45 items in each study. The definition for meaning of items was obtained from the guidelines of original tools and will be compiled and distributed to all reviewers for conducting the scoring. A three point scale (yes, no, unclear), which was the most common response format in the original tools, will be used to assess the items from these tools.

In addition, the Cochrane Collaboration Risk of Bias (RoB) tool introduced in 2008 [20] will be used to evaluate the risk of bias of these trials. Additional information regarding individual trials such as type of interventions and their details such as intensity, frequency, and dosage will be collected. Furthermore, information related to type of outcomes (that is, objective, subjective) and their estimates (that is, mean, SD, sample size), funding source, publication year, design characteristics, statistical analysis, and sample size will be extracted.

A data extraction template hosted in an access program database will be used to store data extraction results.

#### Reviewers

A review panel comprised of five reviewers with experience in different areas of health sciences research will participate in this study.

Reviewer training will be carried out with ten papers not included in the set of papers to be reviewed. Each of the ten training papers will be independently reviewed by all member of the review panel and then discussed by the panel. The first author will perform the training for all raters and will make sure that all reviewers have clarity regarding data extraction.

The two reviewers that assessed the same study will compare their assessments. Any discrepancies will be resolved by discussion between the two reviewers. If a consensus rating would not be achieved, then the two reviewers will consult with a third reviewer (first author) to achieve consensus. Consensus answers will be used for all analyses.

### Objectives 1 and 2: analysis

In order to determine the methodological trial components that affect treatment effect estimates, a two-level analysis will be conducted using a meta-meta-analytic approach with a random-effects model to allow for within and between meta-analyses heterogeneity [26]. The first level analysis (within meta-analysis) will be as follows: the standardized effect size estimates will be obtained for the primary outcome of each trial using the guidelines established by Cohen [27]. The data from each trial will be obtained from each meta-analysis. In the case of studies appearing in more than one review, the study will only be considered once in the meta-analysis with the fewer number of overall studies. For each meta-analysis and each trial component (for example, allocation concealment, randomization, blinding), included studies will be divided in two groups according to the relevant quality item (that is, those adequately addressing the item and those not). Two effect sizes will be calculated for each meta-analysis. The first one corresponding to the pooled effect size from those studies having the characteristic of interest (for example, allocation concealment) and the other for those studies that did not (for example, no or unclear allocation concealment). Thus, for each meta-analysis, the difference between pooled estimates from trials with (for example, allocation concealment) and without the characteristic of interest will be derived. The second level analysis (among meta-analyses) will involve pooling the results of the previous analysis (combined differences from all meta-analyses) to describe the effect of the each trial component across all meta-analyses. The effect sizes will be combined at this stage using the DerSimonian and Laird random effect models [28] to allow for between meta-analysis heterogeneity. STATA statistical software version 12 (College Station, TX: StataCorp LP 4905 Lakeway Drive College Station, Texas 77845–4512 USA) will be used to perform these analyses.

### Objective 3: analysis

Exploratory factor analysis (EFA) will be used to identify the latent structure of the items from the seven PT existing tools. First, the 45 items will be examined to be sure that there was variability across the three response options. Items with no variability will be deleted at this point. The next step will be to estimate the number of common factors. The Kaiser-Guttman rule (number of components with eigenvalues greater than or equal to one yielded by a principal components extraction, the Scree test, and Kaiser’s image factoring followed by varimax rotation will be used to identify the number of common factors that underlay the structure of items [29,30]. Following identification of the number of common items, the items will be subjected to an exploratory factor analysis using principal axis extraction followed by a varimax rotation and an oblique transformation. If the correlations among factors in the oblique solution are low, then the varimax rotation solution would be retained, otherwise the oblique solution. Items that did not load on any of the retained factors will be then sequentially removed. Likewise, items with low factor loadings will be removed (≤ 0.36). SPSS version 17 (SPSS Inc., Chicago, IL, USA) will be used to perform all analyses [29,30]. After conducting the exploratory factor analysis, the retained factors will be named by the first author and then, discussed and verified by the members of the review panel.

### Objectives 4 and 5

#### Identification of items by experts

In order to identify relevant items to evaluate risk of bias of PT trials through experts’ opinion; and to develop guidelines for the design, conduct, reporting, and assessment of RoB of PT RCTs, results of the factor analysis will be considered along with the results of the meta-epidemiological approach (that is, association between methodological characteristics and TE) to make recommendations regarding items to use when assessing risk of bias of PT trials. Results of these analyses will be compiled by the research team. If these two approaches (factor analysis and meta-epidemiological approach) have contradictory results regarding items/factors to be included when evaluating the risk of bias of PT trials, items will be further evaluated by methodological and research experts in PT field. They will provide input for item reduction and selection providing face and content validity evidence to these items.

The input of experts will be obtained through a web-based survey following a Delphi procedure. The Delphi technique has been recognized as an appropriate method when the aim of the study is to reach group consensus from individual expert opinion [31]. The Delphi procedure is an interactive method, suitable for controversial topics such as methodological quality/risk of bias of primary research. It engages experts in the area of interest who are asked to rate a set of items or questionnaire anonymously during consecutive rounds while being provided with the aggregate responses of prior rounds [32].

Items obtained from the analyses (factor analysis and meta-epidemiological approach) and the evidence obtained from the previous studies carried out by our team will be presented to each of the PT experts. Each expert has to choose the most relevant items s/he considers to evaluate the quality/risk of bias for PT RCTs. Each expert will be asked to formulate the following question for each item: 'is this item relevant to evaluate the quality/risk of bias for PT RCTs?’ The answer options will use a nine-point Likert scale (1 strongly disagree, 9 strongly agree), with a score of 5 indicating no opinion or not enough information or experience to judge. Recommendations will be drafted by the research team and will be presented to the expert’s panel in two or three rounds of internet-based surveys using a password-protected Delphi survey instrument.

In addition, personal data such as age, country, place of work and details of research and clinical experience will be collected. It should not take more than 15 minutes to complete all of this activity each time.

#### Experts in PT field

Experts in PT research from different geographical areas (for example, Canada, USA, Australia, France, Switzerland, the Netherlands), will be indentified and contacted by Email and they will be invited to participate. To be considered an expert in the field of PT, the participant has to have the following:

1) Be a physical therapist or has worked in PT research area for at least five years.

2) To have participated in at least one RCT or at least three systematic reviews of interventions in the area of PT.

3) To have knowledge/expertise regarding methodological quality/risk of bias of primary research, specifically RCTs in the area of PT.

In order to have accurate information regarding items/factors that are necessary to evaluate the risk of bias of PT trials, a selected group of experts (with these characteristics) are needed to be able to discern and determine which of the items/factors are relevant to the study question.

### Objectives 4 and 5: analysis

Recommendation-specific medians will be estimated for each of the rounds. The inter-rater agreement between participants in each round will be evaluated using the intraclass correlation coefficient (ICC) with 95% confidence intervals (CI).

Recommendations rated by < 10% of the experts will be discarded. Recommendations rated by 10% to 90% of the experts will be deferred until the second round. Recommendations rated by > 90% of the experts will be discarded if the overall median score fall within the bottom tertile (1 to 3) and deferred until the second round if the overall median score is 4 to 6. Recommendations with overall median scores within the top tertile (7 to 9) will be classified as follows: those rated by < 90% of the experts will be deferred until the second round, and those rated by > 90% of the experts will be classified as 'high agreement’ if they had an overall median score of 9 or as 'agreement’ if they had an overall median score of 7 or 8.

### Data integration

Data from factor analysis, meta-epidemiological approach and Delphi procedure will be integrated by the research team to develop guidelines for the design, conduct, implementation, and reporting of PT RCTs. In addition, guidelines to assess the RoB in PT RCTs will be developed.

## Discussion

### Significance of the proposed research project

The results of this project will make an important contribution to research and practice in the field of PT and the allied health professions. This work will be valuable to a number of stakeholders including researchers, systematic reviewers and meta-analysts, methodologists, clinicians, and policy-makers. The results of the proposed project will be the development and refinement of methods of evidence synthesis for PT, so that the final products are valid and meaningful to the end users. First, this knowledge will provide guidance to researchers for the design, conduct, implementation, and reporting of RCTs in the PT field as well as other similar health areas. Second, the findings of this project will provide clearer benchmarks for evaluating the quality of intervention trials for systematic reviewers and meta-analysts in the area of PT. Third, the information will allow practitioners to better determine the most effective interventions in order to provide their patients with treatment options that are likely to yield greater treatment benefits. Fourth, this information will help policy-makers to make informed decisions regarding implementation and support of PT interventions. The results of this research have important implications for knowledge translation by providing empirical evidence that allows more accurate interpretation of study findings. This is critical for informed decision-making in order to understand the likely effect that therapeutic interventions will have when applied in practice. More informed decision making regarding clinical care based on empirical evidence will ultimately improve patient outcomes and will increase efficiency in the health care system because interventions that are most likely to improve patient outcomes will be taken up and implemented.

### Dissemination plan

We will develop guidelines for the design, conduct, implementation, and reporting of PT trials and guidelines to assess risk of bias in PT RCTs. These products will be disseminated in different ways to a variety of target audiences. Key groups with an interest in evidence based practice in PT such as the Center of Evidence Based Physiotherapy in the Netherlands, the Australian Center for Evidence Based Physiotherapy, the World Confederation for PT Evidence Based Practice team, the Canadian Physiotherapy Association, the Cochrane Bias Methods Group, and the Cochrane Rehabilitation and Related Therapies Field will be engaged. We will disseminate our results to these groups through standard means (for example, technical report, executive summary, peer-reviewed publications) and will also work with these groups to identify tools (for example, synopses, checklists) and methods (for example, list-servs, key meetings and conferences) to distribute the results to a wider audience. Results will also be disseminated to the editors of PT journals. We will also develop innovative methods of training and dissemination such as on-line modules and web-based resources. An advisory working committee will be created to guide implementation of the project findings. Researchers and clinicians in the field will also be targeted using traditional dissemination methods such as presentation at international conferences (for example, International World PT conference), seminars, workshops as well as publications in relevant peer reviewed journals. The findings of this project will be freely available to other researchers and graduate students on request in the form of a final report.

## Abbreviations

EFA: Exploratory factor analysis; MAs: Meta-analyses; PT: Physical therapy; RCTs: Randomized controlled trial(s); RoB: Risk of bias; SRs: Systematic reviews; TENS: Transcutaneous electrical nerve stimulation; TEs: Treatment effects; WCPT: World confederation for physical therapy.

## Competing interest

The authors declare that they have no competing interests.

## Authors’ contributions

Dr. Armijo-Olivo provided concept/idea/research design and writing. Dr. Greta Cummings, Dr. Jorge Fuentes, Dr. Todd Rogers, Dr. Lisa Hartling and Dr. Humam Saltaji provided feedback for concept/idea/research design. All authors critically revised the study protocol and provided final approval of the version to be published.

## Authors’ information

Susan Armijo-Olivo has a BSc in Physical Therapy (PT) from the Pontifical University Catholic of Chile, a MSc PT and a PhD in Rehabilitation Sciences from the University of Alberta. Her major field of research is diagnosis, evaluation, and treatment of patients with musculoskeletal pain especially temporomandibular and cervical spine disorders along with physical therapy evidence based practice, knowledge synthesis and knowledge translation in health research areas. Susan is interested in optimizing and validating methods used to synthesize research and to conduct high quality research particularly in the area of allied health professions. Her post-doctoral project focuses on the methodological predictors of effect size estimates in PT trials. This research is critical to accurately provide conclusions for health care and decision making. This will help disseminate evidence in a useful and understandable way for end-users such as patients, health care clinicians, and policy/decision-makers. This project will be an important contribution to the area of knowledge synthesis and translation in the physical therapy field and allied health professions.
